# Bullying Victimization among In-School Adolescents in Ghana: Analysis of Prevalence and Correlates from the Global School-Based Health Survey

**DOI:** 10.3390/healthcare9030292

**Published:** 2021-03-07

**Authors:** Richard Gyan Aboagye, Abdul-Aziz Seidu, John Elvis Hagan, James Boadu Frimpong, Joshua Okyere, Abdul Cadri, Bright Opoku Ahinkorah

**Affiliations:** 1 School of Public Health, University of Health and Allied Sciences, Ho PMB 31, Ghana; raboagye18@sph.uhas.edu.gh; 2 Department of Population and Health, University of Cape Coast, Cape Coast PMB TF0494, Ghana; abdul-aziz.seidu@stu.ucc.edu.gh (A.-A.S.); joshuaokyere54@gmail.com (J.O.); 3 College of Public Health, Medical and Veterinary Services, James Cook University, Townsville, QSD 4811, Australia; 4 Department of Health, Physical Education, and Recreation, University of Cape Coast, Cape Coast PMB TF0494, Ghana; frimpongboadujames@gmail.com; 5 Neurocognition and Action-Biomechanics-Research Group, Faculty of Psychology and Sport Sciences, Bielefeld University, Postfach 10 10 31, 33501 Bielefeld, Germany; 6 Department of Family Medicine, Faculty of Medicine, McGill University, Montreal, QC H3S 1Z1, Canada; abdul20c@yahoo.com; 7 School of Public Health, Faculty of Health, University of Technology Sydney, Sydney, NSW 2007, Australia; brightahinkorah@gmail.com

**Keywords:** bullying victimization, Ghana, global school-based health survey, in-school adolescents, REBE, social support, suicide ideation

## Abstract

(1) Background: Although bullying victimization is a phenomenon that is increasingly being recognized as a public health and mental health concern in many countries, research attention on this aspect of youth violence in low- and middle-income countries, especially sub-Saharan Africa, is minimal. The current study examined the national prevalence of bullying victimization and its correlates among in-school adolescents in Ghana. (2) Methods: A sample of 1342 in-school adolescents in Ghana (55.2% males; 44.8% females) aged 12–18 was drawn from the 2012 Global School-based Health Survey (GSHS) for the analysis. Self-reported bullying victimization “during the last 30 days, on how many days were you bullied?” was used as the central criterion variable. Three-level analyses using descriptive, Pearson chi-square, and binary logistic regression were performed. Results of the regression analysis were presented as adjusted odds ratios (aOR) at 95% confidence intervals (CIs), with a statistical significance pegged at *p* < 0.05. (3) Results: Bullying victimization was prevalent among 41.3% of the in-school adolescents. Pattern of results indicates that adolescents in SHS 3 [aOR = 0.34, 95% CI = 0.25, 0.47] and SHS 4 [aOR = 0.30, 95% CI = 0.21, 0.44] were less likely to be victims of bullying. Adolescents who had sustained injury [aOR = 2.11, 95% CI = 1.63, 2.73] were more likely to be bullied compared to those who had not sustained any injury. The odds of bullying victimization were higher among adolescents who had engaged in physical fight [aOR = 1.90, 95% CI = 1.42, 2.25] and those who had been physically attacked [aOR = 1.73, 95% CI = 1.32, 2.27]. Similarly, adolescents who felt lonely were more likely to report being bullied [aOR = 1.50, 95% CI = 1.08, 2.08] as against those who did not feel lonely. Additionally, adolescents with a history of suicide attempts were more likely to be bullied [aOR = 1.63, 95% CI = 1.11, 2.38] and those who used marijuana had higher odds of bullying victimization [aOR = 3.36, 95% CI = 1.10, 10.24]. (4) Conclusions: Current findings require the need for policy makers and school authorities in Ghana to design and implement policies and anti-bullying interventions (e.g., Social Emotional Learning (SEL), Emotive Behavioral Education (REBE), Marijuana Cessation Therapy (MCT)) focused on addressing behavioral issues, mental health and substance abuse among in-school adolescents.

## 1. Background

Bullying victimization is one of the major perennial behavioral acts that happen in the school setting [1,2,3]. As such, this aspect of youth violence among in-school adolescents continues to remain a public health issue in most countries around the world, especially for parents, school authorities, and mental health practitioners [2,4,5]. Bullying victimization is both direct or indirect behavioral acts where the perpetrator who is usually powerful abuses the victim verbally (i.e., insulting, teasing, threatening); physically (i.e., coercion, hitting, pushing, kicking, forceful possession of someone’s belongings), or emotionally (i.e., social exclusion, peddling falsehood about someone) in a repeated fashion [6,7,8,9]. According to Vanderbilt and Augustyn [10], these increased risks happen not only to bullies and their victims, but also to bystanders or onlookers. Since its operationalization, bullying victimization through social science research has been associated with an array of adverse health and psychosocial outcomes [11,12,13,14]. These outcomes are not limited to internalizing problems including low self-esteem [15,16], heightened depression [2,17], increased anxiety [2], poor social skills [18,19], suicidal ideations [20] and poor physical health [21]. Additionally, several studies indicate that bullying victimization may cause long-term psychological and/or neurodevelopmental disorders including nightmares, worry, and sadness [4,14,19,22]. Moreover, victims of bullying tend to skip classes more often, perform poorly in academics, avoid school activities, increased substance use, and become truant at a point in time and later develop Learning Disabilities, and Attention Deficit/Hyperactivity Disorder (ADHD) [14,17,23,24,25,26]. Available studies on bullying victimization in low-and-middle-income countries (LMICs) such as Ghana [20], Mozambique [3], Nigeria [27], and Malawi [8] indicate that bullying victimization is still high among in-school adolescents, with socio-demographic (e.g., sex, age, economic status [3,5,9,27,28,29,30,31]) and behavioral characteristics (e.g., loneliness, physical fighting, sexual behaviors, substance use, truancy [2,3,19,31,32]) as its correlates.

Despite the negative short and long-term health and psychological implications of bullying victimization on in-school adolescents, the issue has received less research attention in Ghana, with just a few studies on the concept [20,33]. Our study builds on the study by Acquah, Wilson and Doku [20], which used the 2007 Global School-based Health Survey (GSHS) dataset by using the 2012 version and examined patterns and correlates for bullying among young adolescents in Ghana. Again, whereas the study by Baiden et al. [33] studied bullying victimization as a predictor of suicidal ideation and suicide attempt among adolescents in Ghana, our study focuses on bully victimization, not as a predictor but an outcome of interest. Besides, context-specific influences may differentiate the prevalence and patterns of bullying victimization experiences among adolescents and young adults [34]. Additionally, with many Ghanaian schools not adhering to existing anti-bullying policy, together with previously reported high prevalence of bullying victimization among adolescents, providing contextual updated empirical information on bullying victimization is warranted [35]. Essentially, research on bullying victimization among in-school adolescents in the Ghanaian context is scanty. In order to help with the reduction in the prevalence of bullying victimization in the school settings in Ghana, there is the need for more studies to be carried out to gain an in-depth understanding of the correlates of bullying victimization among Ghanaian in-school adolescents which will, in turn, direct public health actions.

Therefore, the current study examined the prevalence and correlates of bullying victimization among in-school adolescents in Ghana using the 2012 GSHS data. In achieving this objective and based on extant literature (e.g., [3,5,9,18,19,20,21,22]) from LMICs and particularly in Ghana (e.g., [14,24]) on bullying victimization, it was hypothesized that the prevalence rate of bullying victimization would relatively be high. It was further anticipated that bullying victimization among in school adolescents would vary as function of gender, grade level, injury status, whether having or not having physical fights, having or not having suicide attempts and being or not being lonely as well as using or not using a substance [11]. However, because of possible context-specific variations as noted in previous studies, no specific directions were stated regarding the aforementioned hypotheses. Findings from the study could help in the early identification, management, and formulation of anti-bullying policies in Ghanaian schools and also improve already instituted policies.

## 2. Materials and Methods

### 2.1. Study Design, Source of Data, and Sampling Procedure

The data for the study were obtained from the 2012 GSHS of Ghana. The survey was conducted among in-school adolescents from the World Health Organization (WHO) countries to ascertain the risk and protective factors of health risk behaviors. In Ghana, the survey was conducted with partnership from the Center for Disease Control and Prevention (CDC), Middle Tennessee University, WHO, and the Ghana Education Service (GES). A cross-sectional design was employed using a structured questionnaire to obtain data from adolescents. The questionnaire covered health risk behaviors such as alcohol and drug use, dietary behaviors, hygiene, mental health, physical activity, unintentional injury, sexual behavior, tobacco use, violence, and protective factors. A two-stage sampling method was used to recruit adolescents from twenty-five senior high schools in Ghana. At the initial stage, the study schools were selected with probability proportional to the school’s enrolment size. At the last stage, classes within chosen schools were randomly selected and all eligible students were recruited to participate in the study. The ten regions (then) were grouped into three zones; South (Greater Accra, Central, Volta, Eastern), Central (Brong Ahafo, Ashanti, Western) and North (Northern, Upper East and Upper West). Twenty-five (25) schools were randomly selected from each zone making a total of seventy-five schools in all the three zones. In each of the selected school, all the classrooms were included in the sampling frame. All the students in the sampled classrooms were eligible to participate in the survey. However, only students aged 10 to 19 years were studied. The survey included students who were aged 10 to 19 years (period of adolescence), present at school on the day of data collection, and showed evidence of written informed consent (those aged 18 years and above), and written parental or guardian consent form and child assent form (those between 18 years). This sampling method ensured that every eligible student had an equal chance of being selected for inclusion in the study as well as the generalizability of the results to a larger population of in-school adolescents in Ghana. A total of 1984 adolescents from selected senior high schools completed the survey. The school, student, and overall response rates were 96%, 74%, and 71%, respectively. In the present study, a total of 1342 adolescents with complete cases of variables of interest were included in the final analysis. Using this criterion, 66 students who had no information on bullying victimization were excluded from the study. They constituted 4.77% of total number of students eligible for the study. The dataset used is freely available at https://www.who.int/ncds/surveillance/gshs/ghanadataset/en/ (accessed on 6 October 2020). We relied on the “Strengthening the Reporting of Observational Studies in Epidemiology” (STROBE) statement writing the manuscript [36].

### 2.2. Study Variables

#### 2.2.1. Outcome Variable

Bullying victimization was the dependent variable in the study. It was derived from the question “During the past 30 days, on how many days were you bullied?” The possible responses were 1 = 0 days; 2 = 1 or 2 days; 3 = 3 to 5 days; 4 = 6 to 9 days; 5 = 10 to 19 days; 6 = 20 to 29 days; and 7 = All 30 days. For this study, the responses were dichotomized into “Yes” and “No”. Adolescents who reported being bullied at least once during the past 30 days were categorized as “Yes” whilst their counterparts who were not bullied (1 = 0 days) were grouped as “No”. The categorization of the responses was informed by literature [2,3].

#### 2.2.2. Explanatory Variables

The explanatory variables used in the study were based on the associations established in previous literature (e.g., [3,37]) and most importantly, their availability in the GSHS dataset. The variables consisted of socio-demographic (age, sex, grade, and hunger), health risk behaviors (physical fighting, physical attack, injury, truancy, alcohol use, cigarette smoking, marijuana use, tobacco use, suicidal ideation, suicidal attempt, loneliness, anxiety, and bullied), and protective factors (peer support, close friends, parental or guardian supervision, parental or guardian bonding, and parental or guardian connectedness). The variables we studied have been validated and used in several previous studies [2,3,8,31,33,37,38]. The detailed description of the variables and the recoded responses have been shown in Table 1.

### 2.3. Statistical Analyses

The data analysis was carried out using Stata software version 16.0 (Stata Corporation, College Station, TX, USA). Three levels of analyses consisting of descriptive, Pearson chi-square, and binary logistic regression were performed. Descriptively, frequencies, and percentages were used to present the prevalence of the categorical variables. Secondly, Pearson chi-square test was carried out to determine the association between bullying victimization (outcome variable) and explanatory variables. The variables that showed a significant association (*p* < 0.05) were further placed in the logistic regression. At the binary logistic regression analysis, three models were used. The first model (Model I) was built to determine the association between socio-demographic variables (sex, grade, and hunger) and bullying victimization that were significant at the chi-square analysis. The second model (Model II) was built to examine the association between socio-demographic variables and health risk behaviors. The last model (Model III) was fitted to determine the association between all the explanatory variables that established a significant association at the chi-square analysis and bullying victimization. The results of the regression analyses were presented as adjusted odds ratios (aOR) with their respective 95% confidence intervals (CIs). All the recoded variables and reference categories used were determined based on the review of pertinent literature [2,35,38]. A p-value of less than 5% was considered significant. We checked for multicollinearity with the Variance Inflation Factor (VIF). The result showed a mean, minimum, and maximum VIFs were 1.26, 1.04, and 1.62, respectively, indicating no evidence of multicollinearity.

### 2.4. Ethical Issues

Ethical requirements for the use of both minors and non-minors were adhered to during the survey. First, institutional permission for the conduct of the study was sought from the Ministry of Education and Heads of selected schools. Secondly, written informed consent was sought from students 18 years and above before inclusion in the study. For those below 18 years, written child assent and parental or guardian consent forms were obtained from each child before participating in the study. The adolescents anonymously and voluntarily completed the survey questionnaire.

## 3. Results

### 3.1. Prevalence and Bivariate Analysis of Bullying Victimization among In-School Adolescents in Ghana

Table 2 presents the prevalence and bivariate analysis of bullying victimization among in-school adolescents in Ghana. The prevalence of bullying victimization among in-school adolescents in Ghana was 41.3%. We had significant differences in the experience of bullying victimization across sex, grade, ever went hungry, experience of injury, fight, attack, anxiety, loneliness, suicidal ideation, suicidal attempt, alcohol use, tobacco use, marijuana use, peer support and parental or guardian respect for privacy. All variables that showed significant differences had a *p*-value < 0.05. Bullying victimization was more prevalent in females (44.3%), adolescents in SHS 2 (54.2%), in-school adolescents who ever went hungry (50.3%), injured (55.1%), engaged in fighting (60.8%), attacked (56.3%), felt anxious (51.6%), felt lonely (55.0%), had suicidal ideation (57.2%), and attempted suicide (61.7%). In the area of substance use, bullying victimization was prevalent among in-school adolescents who were current alcohol users (56.1%), current tobacco users (73.0%), current cigarette smoking (74.4%), and current marijuana users (84.4%). Additionally, bullying victimization was more prevalent among in-school adolescents with without peer support (37.3%) and those with parental or guardian respect for privacy (51.4%).

### 3.2. Multivariable Regression Analysis of Predictors of Bullying Victimization among In-School Adolescents in Ghana

Table 3 presents the results of the multivariable logistic regression. It was realized in the final model (Model III) that adolescents in SHS 3 and SHS 4 were less likely to be victims of bullying. Adolescents who had sustained injury were more likely to be bullied compared to those who had not sustained any injury. The odds of bullying victimization were higher among adolescents who had engaged in physical fight and those who had been physically attacked. Additionally, adolescents who felt lonely were more likely to report being bullied as against those who did not feel lonely. Additionally, adolescents with a history of suicide attempts were more likely to be bullied. Similarly, adolescents who used marijuana had higher odds of experiencing bullying victimization.

## 4. Discussion

Drawing from a nationally representative dataset, that is, the 2012 GSHS data, the study examined the prevalence and correlates of bullying victimization in Ghana. As hypothesized in the current study, the prevalence of bullying victimization among in-school adolescents in Ghana (41.3%) was relatively higher compared to what was found by Baiden et al. [33] (40%) but lower than the 60% prevalence that was found by Acquah et al. [20]. The possible reason for the difference in the findings could be the different survey years and sample size as well as structural variations in school settings, communities, and cultures [2]. Overall, socio-demographic factors demonstrated some variations on their association with bullying victimization, though partly as hypothesized.

Specifically, increasing grade was associated with lower odds of being bullied among the in-school adolescents in Ghana. This association confirms previous studies that bullying victimization decreases with increasing educational grade [39,40,41,42]. A plausible reason for the observed association could be that those in senior levels (SHS 3 and 4) were more physically and psychologically developed to protect themselves from being bullied compared those in lower levels [39]. Additionally, Owusu et al. [35] hypothesized in their study that among senior high schools in Ghana, lower levels of bullying victimization among those in higher levels could be as a result of the older aged students in those levels. Hence, students in higher levels in educational grade especially in Ghana are regarded as “seniors” and sometimes perpetuators of bullying rather than being victims.

Again, adolescents who used marijuana had a higher likelihood of being bullied. A growing body of national and international research suggests that all types of bullying victimization create a proximal risk for substance misuse and/or abuse among adolescents [43]. Current finding is corroborated by several studies [44,45] that showed that substance abuse among adolescents increases their odds of experiencing bullying victimization. Although literature is replete with findings that drug/substance abuse exacerbates the odds of being bullied as an adolescent, most of these studies focused mainly on tobacco and alcohol use (e.g., [20,44]). The association between marijuana use and bullying victimization could also be rooted in Lazarus’ coping theory [46]. This theory postulates that adolescents may engage in risk health-compromising behaviors (e.g., marijuana use) as a coping response to the exposure of unbearable life stressors such as bullying victimization. Therefore, current finding that marijuana use increases the odds of bullying victimization sets the tone for future studies to further replicate this association.

Suicidal attempts have been shown to be significantly associated with bullying victimization [47,48]. Our study confirmed these earlier findings by showing that greater odds of being bullied occurred among in-school adolescents who reported attempted suicidal behaviors. This finding epitomizes that not only does the suicidal attempts predict bullying victimization; the reverse can be the case where bullying victimization exacerbates the tendency to attempt suicide. According to Opperman et al. [49], bullying victimization has a high potential to create a deep sense of not belonging and/or feelings of unwantedness which tends to increase the odds of attempting suicide. Moreover, bullying victimization exacerbates low self-esteem [50] and diminished self-worth [51], resulting in suicidal ideations and subsequent suicide attempts. Some scholars have argued that the concept of impulsivity may also explain why some in-school adolescents may engage in violent and other risk-taking behaviors; suicidal behaviors [52,53] and substance use [54] because of sensation seeking [33,55]. Although available literature has focused on bullying victimization as the predictor of suicidal attempts, our findings reveal that suicidal attempt also predicts the odds or likelihood for an adolescent in school to experience bullying victimization.

Physically attacked and being exposed to injury increased the likelihood for adolescents in schools to be bullied. This finding is supported by Acquah, Wilson and Doku [20] who also found that being physically attacked and being exposed to injury was associated with higher odds of bullying victimization. Although the direct pathway between physical attack, injury and bullying victimization remains unclear, our findings is explainable from the perspective that being repeatedly attacked physically in itself constitutes bullying and the concomitant injuries can be a precursor or consequence of bullying [9].

Other results show that being lonely increases the odds of being bullied. The finding is consistent with the results from Campbell [56] who found that feelings of hopelessness and loneliness was more likely to be reported by adolescents who have been victims of all kinds of bullying. Adolescence is a transitional phase of human life and therefore, adolescents yearn to belong and associate. However, when this is not achieved but rather replaced with loneliness, it subjects the adolescents to all forms of bullying that can even exacerbate the loneliness being faced [57].

### 4.1. Strength and Limitations

The use of a nationally representative dataset like the GSHS is a major strength for our study as it allows us to be able to generalize the findings to in-school adolescents in Ghana. Moreover, the survey questionnaire has been used and validated across different cultural settings. Notwithstanding, our study had some limitations. We relied on data from a cross-sectional survey and for that matter, only associations can be made; causality cannot be made. Secondly, the use of a secondary data limited our analysis only to variables that were available in the dataset. Additionally, bullying victimization was assessed using only one question “During the past 30 days, on how many days were you bullied?” Hence, this criterion may affect the accuracy and reliability of the findings. Additionally, since the survey was a self-reported one, it can be subjected to non-response bias and social desirability. Therefore, other key theoretical predictors such as the effects of bystanders could not be accounted for or included in our analysis. As such, our results should be interpreted in light of the aforementioned limitations.

### 4.2. Practical Implications for School Health

School-based anti-bullying programs should address the burden of bullying victimization at different grade levels to better mirror each sub-group level experiences. Other school-based interventions should take into account that among in-school adolescents, especially males, physical forms of bullying victimization (e.g., physical fights), being attacked, adolescents who demonstrate elements of loneliness, those with hunger related challenges, and exhibit potential suicide attempts. Policy makers (e.g., Ghana Education Service) in conjunction school authorities should effectively revitalize existing school-based anti-bullying interventions and potential new programs through appropriate curriculum planning to promote positive school climate. Programs such as regular anti-bullying screening of adolescents in schools to ensure prevention, identification and early management to reduce adverse impacts connected with academic (e.g., school dropouts) and health outcomes (e.g., mental and emotional disorders) are encouraged. Other interventions (e.g., Social Emotional Learning [SEL], Rational Emotive Behavioral Education [REBE], peer social network systems) should help strengthen in-school adolescents’ resilience, improve their social environments, and foster positive youth development.

## 5. Conclusions

Current findings show that four in every ten in-school adolescents in Ghana had experienced bullying victimization in the past 30 days prior to the study. This finding further reiterates the earlier assertion that bullying victimization is quite pervasive among in-school adolescents in the country. Factors identified to be associated with bullying victimization were grade of the student, engaging in physical fights, being attacked physically, injury, loneliness, suicidal attempts, and current use of marijuana. There is the need for policy makers and school authorities in Ghana to design and implement policies and anti-bullying interventions focused on addressing behavioral issues, mental health and substance abuse that can results in bullying among in-school adolescents as cited earlier. Future school context-specific studies could investigate what other specific factors (e.g., student-teacher ratio, school density, teacher attributes) not captured in the current study might influence bullying victimization.

### Declarations

Ethics Approval and Consent to Participate: The survey was conducted with strict adherence to ethical protocols. In various countries, institutional permission was sought from either the Ministry of Education or the Ministry of Health. All the ethical requirements from these institutions were strictly adhered to, especially, concerning the inclusion of minors in a study. At the school level, written informed consent was sought from the heads of various schools included in the study. For adolescents below 18 years, parental or guardian consent and child assent were sought from them before inclusion into the study. Additionally, written informed consent was obtained from those aged 18 years or older. The sampled students anonymously and voluntarily completed the survey questionnaire.

## Figures and Tables

**Table 1:** Study Variables.

Variables	Question	Response Options and Recoding
**Outcome Variable**		
Bullied	During the past 30 days, on how many days were you bullied?	1 = 0 days; 2 = 1 or 2 days; 3 = 3 to 5 days; 4 = 6 to 9 days; 5 = 10 to 19 days; 6 = 20 to 29 days; and 7 = All 30 days (coded as 1 = No; and 2–7 = Yes)
**Explanatory Variables**		
**Socio-Demographic Characteristics**		
Age	How old are you?	1 = 12, 2 = 13, 3 = 14, 4 = 15, 5 = 16, 6 = 17, 7 = 18 years (coded as 11–14 = 0; 15–18 = 1)
Sex	What is your sex?	1 = male, 2 = female (coded 2 = 0, 1 = male)
Grade	In what grade are you?	1 = SHS1, 2 = SHS2, 3 = SHS3, 4 = SHS4
Hunger (proxy of socioeconomic status)	During the past 30 days, how often did you go hungry because there was not enough food in your home?	1 = never, 2 = Rarely, 3 = sometimes, 4 = most of the times, 5 = always (coded 1–3 = No; 4–5 = Yes)
**Psychosocial Environmental Factors**		
Tobacco use	During the past 30 days, on how many days did you use any other form of tobacco, such as chewing tobacco leaves?	1 = 0 days; to 7 = All 30 days (coded as 1 = No; and 2–7 = Yes)
Alcohol use	During the past 30 days, on how many days did you have at least one drink containing alcohol?	1 = 0 days; to 7 = All 30 days (coded as 1 = No; and 2–7 = Yes)
Smoking	During the past 30 days, how many days did you smoke cigarette?	1 = 0 days; to 7 = All 30 days (coded as 1 = No; and 2–7 = Yes)
Marijuana use	During the past 30 days, how many times have you used marijuana (also called weed, Jah, Indian hemp, ahabammono, and ganja)	1 = 0 times; to 5 = 20 or more times (coded as 1 = No; and 2–5 = Yes)
Loneliness	During the past 12 months, how often have you felt lonely?	1 = never, 2 = rarely, 3 = sometimes, 4 = most of the time to 5 = always (coded as 1–3 = No; and 4–5 = Yes)
Anxiety	During the past 12 months, how often have you been so worried about something that you could not sleep at night?	1 = never, 2 = Rarely, 3 = sometimes, 4 = most of the times, 5 = always (coded 1–3 = No, 4–5 = Yes)
Injury	During the past 12 months, how many times were you seriously injured?	1 = 0 times; to 8 = 12 or more time (coded as 1 = No; and 2–8 = Yes)
Truancy	During the past 30 days, on how many days did you miss classes or school without permission?	1 = 0 days, 2 = 1 or 2 days, 3 = 3 to 5 days, 4 = 6 to 9 days, 5 = 10 or more (coded as 1 = No; and 2–5 = Yes)
Suicidal ideation	During the past 12 months, did you ever seriously consider attempting suicide?	1 = yes, 2 = no (coded 2 = No; and 1 = Yes)
Suicidal attempt	During the past 12 months, how many times did you actually attempt suicide?	1 = 0 times; 2 = 1 time; 3 = 2 or 3 times; 4 = 4 or 5 times; and 5 = 6 or more times (coded as 1 = No; and 2–5 = Yes)
Fight	During the past 12 months, how many times were you in a physical fight?	1 = 0 times; 2 = 1 time; 3 = 2 or 3 times; 4 = 4 or 5 times; 5 = 7 or 7 times; 6 = 8 or 9 times; 7 = 10 or 11 times; and to 8 = 12 or more times (coded as 1 = No; and 2–8 = Yes)
Injury	During the past 12 months, how many times were you seriously injured?	1 = 0 times; 2 = 1 time; 3 = 2 or 3 times; 4 = 4 or 5 times; 5 = 7 or 7 times; 6 = 8 or 9 times; 7 = 10 or 11 times; and to 8 = 12 or more times (coded as 1 = No; and 2–8 = Yes)
Attacked	During the past 12 months, how many times were you physically attacked?	1 = 0 times; 2 = 1 time; 3 = 2 or 3 times; 4 = 4 or 5 times; 5 = 7 or 7 times; 6 = 8 or 9 times; 7 = 10 or 11 times; and to 8 = 12 or more times (coded as 1 = No; and 2–8 = Yes)
Close friends	How many close friends do you have?	1 = 0; 2 = 1; 3 = 2; and 4 = 3 or more (coded as 1 = No; and 2–4 = Yes)
Helpful(Peer support)	During the past 30 days, how often were most of the students in your school kind and helpful?	1 = never, 2 = Rarely, 3 = sometimes, 4 = most of the times, 5 = always (coded as 1–3 = No; and 4–5 = Yes)
Parents check homework (parental supervision)	During the past 30 days, how often did your parents or guardians check to see if your homework was done?	1 = never, 2 = Rarely, 3 = sometimes, 4 = most of the times, 5 = always (coded as 1–3 = No; and 4–5 = Yes)
Understand problems (Parental Connectedness)	During the past 30 days, how often did your parents or guardians understand your problems and worries?	1 = never, 2 = Rarely, 3 = sometimes, 4 = most of the times, 5 = always (coded as 1–3 = No; and 4–5 = Yes)
Know what adolescent do free time (Parental or guardian Bonding)	During the past 30 days, how often did your parents or guardians really know what you were doing with your free time?	1 = never, 2 = Rarely, 3 = sometimes, 4 = most of the times, 5 = always (coded as 1–3 = No; and 4–5 = Yes)
Parental or guardian respect for Privacy	During the past 30 days, how often did your parents or guardians go through your things without your approval?	1 = never, 2 = Rarely, 3 = sometimes, 4 = most of the times, 5 = always (coded as 1–3 = No; and 4–5 = Yes)

**Table 2:** Bivariate Analysis of the Proportion of Bullying Victimization among In-school Adolescents in Ghana.

	N = 1342		Bullying Victimization	Chi-Square χ^2^ (*p*-Value)
Variable	Frequency	Percentage	Yes (41.3%)	
**Age**				1.23 (0.268)
12–14 years	38	2.8	50.0	
15–18 years	1304	97.2	41.0	
**Sex**				3.98 (0.046)
Female	601	44.8	44.3	
Male	741	55.2	38.9	
**Grade**				85.98 (<0.001)
SHS 1	396	29.5	54.0	
SHS 2	253	18.8	54.2	
SHS 3	429	32.0	30.8	
SHS 4	264	19.7	26.9	
**Ever went hungry**				6.23 (0.013)
No	1179	87.9	40.0	
Yes	163	12.1	50.3	
**Injury**				112.92 (<0.001)
No	651	48.5	26.4	
Yes	691	51.5	55.1	
**Engaged in fight**				80.38 (<0.001)
No	972	72.4	33.8	
Yes	370	27.6	60.8	
**Attacked**				70.56 (<0.001)
No	857	63.9	32.8	
Yes	485	36.1	56.3	
**Truant**				1.81 (0.179)
No	933	69.5	40.1	
Yes	409	30.5	44.0	
**Anxiety**				9.51 (0.002)
No	1156	86.1	39.6	
Yes	186	13.9	51.6	
**Loneliness**				22.78 (<0.001)
No	1100	82.0	38.3	
Yes	242	18.0	55.0	
**Suicidal ideation**				25.77 (<0.001)
No	1134	84.5	38.4	
Yes	208	15.5	57.2	
**Suicidal attempt**				55.65 (<0.001)
No	1081	80.6	36.4	
Yes	261	19.4	61.7	
**Current alcohol use**				15.94 (<0.001)
No	1187	88.5	39.3	
Yes	155	11.5	56.1	
**Current tobacco use**				27.46 (<0.001)
No	1279	95.3	39.7	
Yes	63	4.7	73.0	
**Current cigarette smoking**				18.13 (<0.001)
No	1303	97.1	40.3	
Yes	39	2.9	74.4	
**Current marijuana use**				25.11 (<0.001)
No	1310	97.6	40.2	
Yes	32	2.4	84.4	
**Close friends**				3.16 (0.076)
No	179	13.3	35.2	
Yes	1163	86.7	42.2	
**Peer support**				4.79 (0.029)
No	870	64.8	43.4	
Yes	472	35.2	37.3	
**Parental or guardian supervision**				2.97 (0.085)
No	776	57.8	39.3	
Yes	266	42.2	44.0	
**Parental or guardian connectedness**				2.17 (0.141)
No	731	54.5	43.1	
Yes	611	45.5	39.1	
**Parental or guardian bonding**				0.11 (0.741)
No	802	59.8	41.6	
Yes	540	40.2	40.7	
**Parental or guardian respect for privacy**				10.67 (0.001)
No	1130	84.2	39.4	
Yes	212	15.8	51.4	

Source: GSHS, 2012.

**Table 3:** Multivariable Analysis of Predictors of Bullying Victimization among In-school Adolescents in Ghana.

	Bullying Victimization		
Variable	Model I aOR [95% CI]	Model II aOR [95% CI]	Model III aOR [95% CI]
**Sex**			
Female	Ref.	Ref.	Ref.
Male	0.78 * [0.62, 0.97]	0.80 [0.63, 1.03]	0.82 [0.64, 1.05]
**Grade**			
SHS 1	Ref.	Ref.	Ref.
SHS 2	0.97 [0.71, 1.34]	0.92 [0.65, 1.30]	0.90 [0.64, 1.27]
SHS 3	0.37 *** [0.28, 0.49]	0.35 *** [0.25, 0.48]	0.34 *** [0.25, 0.47]
SHS 4	0.31 *** [0.22, 0.43]	0.30 *** [0.21, 0.43]	0.30 *** [0.21, 0.44]
**Ever went hungry**			
No	Ref.	Ref.	Ref.
Yes	1.63 * [1.15, 2.29]	1.41 [0.96, 2.06]	1.39 [0.95, 2.03]
**Injury**			
No		Ref.	Ref.
Yes		2.12 *** [1.64, 2.74]	2.11 *** [1.63, 2.73]
**Engaged in fight**			
No		Ref.	Ref.
Yes		1.92 *** [1.45, 2.56]	1.90 *** [1.42, 2.52]
**Attacked**			
No		Ref.	Ref.
Yes		1.72 *** [1.32, 2.25]	1.73 *** [1.32, 2.27]
**Anxiety**			
No		Ref.	Ref.
Yes		1.16 [0.81, 1.66]	1.15 [0.80, 1.65]
**Loneliness**			
No		Ref.	Ref.
Yes		1.52 * [1.10, 2.11]	1.50 * [1.08, 2.08]
**Suicidal ideation**			
No		Ref.	Ref.
Yes		0.93 [0.62, 1.40]	0.94 [0.62, 1.41]
**Suicidal attempt**			
No		Ref.	Ref.
Yes		1.66 * [1.13, 2.42]	1.63 * [1.11, 2.38]
**Current alcohol use**			
No		Ref.	Ref.
Yes		1.34 [0.90, 2.01]	1.33 [0.88, 1.99]
**Current tobacco use**			
No		Ref.	Ref.
Yes		1.49 [0.74, 3.00]	1.43 [0.71, 2.89]
**Current smoking**			
No		Ref.	Ref.
Yes		1.19 [0.46, 3.06]	1.21 [0.47, 3.10]
**Current marijuana use**			
No		Ref.	Ref.
Yes		3.30 * [1.09, 10.03]	3.36 * [1.10, 10.24]
**Peer support**			
No			Ref.
Yes			0.82 [0.63, 1.06]
**Parental or guardian respect for privacy**			
No			Ref.
Yes			1.20 [0.85, 1.68]
N	1342	1342	1342
Pseudo R^2^	0.0544	0.1633	0.1650

Source: GSHS, 2012. aOR: Adjusted Odds Ratio; CI: Confidence interval, Ref: Reference category * *p* < 0.05 *** *p* < 0.001.

## Data Availability

Publicly available dataset was analyzed in this study. This data can be found here: https://www.who.int/ncds/surveillance/gshs/ghanadataset/en/ (accessed on 6 October 2020).
